# Stunting Following Moderate-to-Severe Diarrhea Among Children Aged <5 Years in Africa Before and After Rotavirus Vaccine Introduction: A Comparison of the Global Enteric Multicenter Study and the Vaccine Impact on Diarrhea in Africa (VIDA) Study

**DOI:** 10.1093/cid/ciac948

**Published:** 2023-04-19

**Authors:** Dilruba Nasrin, Yuanyuan Liang, Jennifer R Verani, Helen Powell, Samba O Sow, Richard Omore, M Jahangir Hossain, Sanogo Doh, Syed M A Zaman, Joquina Chiquita M Jones, Alex O Awuor, Irene N Kasumba, Sharon M Tennant, Usha Ramakrishnan, Karen L Kotloff

**Affiliations:** Department of Medicine, Center for Vaccine Development and Global Health, University of Maryland School of Medicine, Baltimore, Maryland, USA; Department of Epidemiology and Public Health, University of Maryland School of Medicine, Baltimore, Maryland, USA; Division of Global Health Protection, US Centers for Disease Control and Prevention, Nairobi, Kenya; Department of Pediatrics, Center for Vaccine Development and Global Health, University of Maryland School of Medicine, Baltimore, Maryland, USA; Department of Medicine, Center for Vaccine Development and Global Health, University of Maryland School of Medicine, Baltimore, Maryland, USA; Centre pour le Développement des Vaccins du Mali, Bamako, Mali; Kenya Medical Research Institute, Center for Global Health Research, Kisumu, Kenya; Medical Research Council Unit The Gambia at the London School of Hygiene & Tropical Medicine, Banjul, The Gambia; Centre pour le Développement des Vaccins du Mali, Bamako, Mali; Medical Research Council Unit The Gambia at the London School of Hygiene & Tropical Medicine, Banjul, The Gambia; Medical Research Council Unit The Gambia at the London School of Hygiene & Tropical Medicine, Banjul, The Gambia; Kenya Medical Research Institute, Center for Global Health Research, Kisumu, Kenya; Department of Medicine, Center for Vaccine Development and Global Health, University of Maryland School of Medicine, Baltimore, Maryland, USA; Department of Medicine, Center for Vaccine Development and Global Health, University of Maryland School of Medicine, Baltimore, Maryland, USA; Hubert Department of Global Health, Rollins School of Public Health, Emory University, Atlanta, Georgia, USA; Department of Medicine, Center for Vaccine Development and Global Health, University of Maryland School of Medicine, Baltimore, Maryland, USA; Department of Epidemiology and Public Health, University of Maryland School of Medicine, Baltimore, Maryland, USA; Department of Pediatrics, Center for Vaccine Development and Global Health, University of Maryland School of Medicine, Baltimore, Maryland, USA

**Keywords:** diarrhea, stunting, children, GEMS, VIDA

## Abstract

**Background:**

Studies conducted before rotavirus vaccine introduction found that moderate-to-severe diarrhea (MSD) in children aged <5 years was associated with stunting at follow-up. It is unknown whether the reduction in rotavirus-associated MSD following vaccine introduction decreased the risk of stunting.

**Methods:**

The Global Enteric Multicenter Study (GEMS) and the Vaccine Impact on Diarrhea in Africa (VIDA) study, two comparable matched case-control studies, were conducted during 2007–2011 and 2015–2018, respectively. We analyzed data from 3 African sites where rotavirus vaccine was introduced after GEMS and before starting VIDA. Children with acute MSD (<7 days onset) were enrolled from a health center and children without MSD (diarrhea-free for ≥7 days) were enrolled at home within 14 days of the index MSD case. The odds of being stunted at a follow-up visit 2–3 months after enrollment for an episode of MSD was compared between GEMS and VIDA using mixed-effects logistic regression models controlling for age, sex, study site, and socioeconomic status.

**Results:**

We analyzed data from 8808 children from GEMS and 10 579 from VIDA. Among those who were not stunted at enrollment in GEMS, 8.6% with MSD and 6.4% without MSD became stunted during the follow-up period. In VIDA, 8.0% with MSD and 5.5% children without MSD developed stunting. An episode of MSD was associated with higher odds of being stunted at follow-up compared with children without MSD in both studies (adjusted odds ratio [aOR], 1.31; 95% confidence interval [CI]: 1.04–1.64 in GEMS and aOR, 1.30; 95% CI: 1.04–1.61 in VIDA). However, the magnitude of association was not significantly different between GEMS and VIDA (*P* = .965).

**Conclusions:**

The association of MSD with subsequent stunting among children aged <5 years in sub-Saharan Africa did not change after rotavirus vaccine introduction. Focused strategies are needed for prevention of specific diarrheal pathogens that cause childhood stunting.

## Teaser

Despite reductions in the attributable burden of rotavirus following vaccine introduction, the association between moderate to severe diarrhea and stunting at a 2–3 month follow-up did not change between studies conducted before and after rotavirus vaccine introduction at 3 sub-Saharan African sites.

The Global Enteric Multicenter Study (GEMS) was a prospective, age-stratified, matched, case-control study of the incidence, etiology, and adverse clinical consequences of moderate-to-severe diarrhea (MSD) in children aged <5 years that was conducted during 2007–2011 at 7 Asian and African sites [1, 2]. The study found rotavirus to be the leading cause of MSD in children. Rotavirus had the largest attributable fraction (AF) of any pathogen at all sites during infancy. Although the AF generally decreased with age, rotavirus continued to have the largest AF of any pathogen in children aged 12–23 months at 4 sites and at the 2 sites in the 24–59 month age group.

Children with MSD in GEMS experienced significantly more linear growth faltering during the 2–3 months following enrollment than their matched controls despite having comparable growth metrics at baseline [2]. Specific pathogens, including *Cryptosporidium*, *Shigella* episodes not treated with World Health Organization (WHO) antibiotics recommended for dysentery, enterotoxigenic *Escherichia coli* encoding heat-stable toxin (ST-ETEC), and typical enteropathogenic *E. coli* (EPEC) were significantly associated with growth faltering during 2–3 months of follow-up in an analysis of MSD cases aged 0–23 months [3]. These findings suggest the hypothesis that pathogens other than rotavirus led to growth faltering following an episode of MSD. Therefore, reducing the AF of rotavirus by introducing rotavirus vaccine would not be expected to weaken the association between MSD and growth faltering.

To examine the impact of rotavirus vaccine introduction on the relationship between diarrheal illness and stunting, we compared the association between MSD-associated growth faltering in GEMS and a subsequent study that used methods comparable to those used in GEMS: the Vaccine Impact on Diarrhea in Africa (VIDA) study. VIDA was conducted at 3 GEMS sites in sub-Saharan Africa where rotavirus vaccine had been introduced after GEMS was completed.

## METHODS

### Study Design

The VIDA study was a prospective, age-stratified, population-based, matched, case-control study that was initiated following rotavirus vaccine introduction at 3 GEMS sites in sub-Saharan Africa: Basse and Bansang, The Gambia; Bamako, Mali; and Siaya County, Kenya. Each site maintained a censused population with an ongoing demographic surveillance system (DSS) from which cases and controls were enrolled. Details of the study design described previously [4] are summarized below.

Participants from 3 age strata (0–11, 12–23, and 24–59 months) seeking care at sentinel health centers serving the DSS population were enrolled between 2007 and 2011 in GEMS and between 2015 and 2018 in VIDA. MSD was defined as ≥3 abnormally loose stools within the previous 24 hours that started within the previous 7 days following ≥7 diarrhea-free days in a child aged <60 months with at least 1 of the following: dehydration (sunken eyes, decreased skin turgor, or intravenous rehydration prescribed), dysentery (visible blood in stools by caretaker report or study team observation), or hospitalization recommended. We enrolled 1–3 controls (without diarrhea in the previous 7 days) per case from the community within 14 days of the index case who were randomly selected from the DSS database and matched to the index case by age, sex, and residence [1].

### Data Collection

At enrollment, caretakers of cases and controls provided sociodemographic, epidemiological, and clinical information during a standardized interview. Child age and rotavirus vaccination status were ascertained from source documents, including birth certificates, vaccination cards, and health center registers. Caretakers were taught to use a pictorial Memory Aid card to record whether the child had diarrhea on each of 14 days following enrollment [1]. The study team visited each case and control at home 2–3 months (50–90 days) after enrollment to assess the vital status and interim health events, perform anthropometric measurements, and collect the Memory Aid.

Training, assessment of competency, and oversight of staff in anthropometry has been described [5]. Staff measured length/height at enrollment and follow-up. Using a Shorr board, standing height was measured for children aged ≥2 years, while the length of children aged <2 years was measured supine. Length/height of each child was measured 3 times to the nearest 0.1 cm, and the median was calculated for analysis [5]. Measurements were repeated if any of the 3 measurements deviated by >0.5 cm from other measurements.

### Statistical Methods

#### Variable Definitions

Age, measured on a continuous scale, was analyzed using the study strata (0–11, 12–23, and 24–59 months). Length/height-for-age *z* score (HAZ) was calculated based on WHO age-specific standards [6, 7]. Because of high malnutrition prevalence at the study sites, we modified WHO 2006 cleaning criteria for exclusionary (implausible) HAZ values to allow for broader deviations from the mean (HAZ <−6 or >6 and a change of HAZ >3 within the follow-up period). The WHO definition of stunting (HAZ <−2.0) [8] at follow-up was used as the outcome. Indicator variables of socioeconomic status (SES) are shown in Table 1. We created an estimated continuous score for household possession of electricity, television, and refrigerator, called the “ETR score,” using principal component analysis for each study [9]. Both water and sanitation were categorized based on source or facility used as improved or unimproved using predefined criteria [10]. Duration of diarrhea was defined as the child's number of diarrhea days beginning 7 days before enrollment until the 14 days post-enrollment as reported in the Memory Aid. If a child was diarrhea-free at enrollment but developed diarrhea within 14 days following enrollment, this was captured by this variable. Seven or more diarrhea-free days indicated the episode ended, and no additional days were counted.

**Table 1.** Comparison of Demographic, Socioeconomic, and Clinical Characteristics of Children in the Global Enteric Multicenter Study and the Vaccine Impact on Diarrhea in Africa Study

Characteristic	Global Enteric Multicenter Study (n = 8808)	Vaccine Impact on Diarrhea in Africa Study (n = 10 579)	*P* Value
**Demographic features**			
ȃAge, n (%), m			
ȃȃ0–11	3283 (37.3)	3664 (34.6)	**<**.**0001**
ȃȃ12–23	3042 (34.5)	3656 (34.6)	
ȃȃ24–59	2483 (28.2)	3259 (30.8)	
ȃSite, n (%)			
ȃȃThe Gambia	2154 (24.5)	3625 (34.3)	**<**.**001**
ȃȃMali	3567 (40.5)	3438 (32.5)	
ȃȃKenya	3087 (35.0)	3516 (33.2)	
ȃFemale, n (%)	3883 (44.1)	4903 (46.4)	.**002**
>2 children aged <5 years children per caretaker, n (%)	4138 (47.0)	5076 (48.0)	.16
Crowding (>3 people sleeping per room), n (%) (missing = 2)	3761 (42.7)	4267 (40.3)	.**001**
**Socioeconomic features**			
ȃCaretaker completed primary school or higher, n (%)	2296 (26.1)	3656 (34.6)	**<**.**001**
Household possessions			
ȃCar, n (%) (missing = 8)	1069 (12.1)	1234(11.7)	.321
ȃScooter, n (%) (missing = 8)	3599 (40.9)	5294 (50.1)	**<**.**0001**
ȃBike, n (%) (missing = 8)	5253 (59.6)	5694 (53.9)	**<**.**0001**
ȃCart, n (%) (missing = 8)	1961 (22.3)	2923 (27.7)	**<**.**0001**
ȃPhone, n (%) (missing = 8)	7255 (82.4)	10 176 (96.3)	**<**.**0001**
ȃRadio, n (%) (missing = 8)	7536 (85.6)	8195 (77.5)	**<**.**0001**
ȃAgricultural land, n (%) (missing = 8)	4521 (51.3)	6211 (58.8)	**<**.**0001**
ȃElectricity, n (%) (missing = 8)	3926 (44.6)	5705 (54.0)	**<**.**0001**
ȃTelevision, n (%) (missing = 8)	3807 (43.2)	4839 (45.8)	.**0004**
ȃRefrigerator, n (%) (missing = 8)	1208 (13.7)	1675 (15.9)	**<**.**0001**
Principal component Electricity, television, refrigerator, mean (SD)	0 (1.45)	0 (1.44)	1
ȃFinished floor, n (%) (missing = 21)	5898 (67.0)	7678 (72.7)	**<**.**0001**
ȃClean cooking fuel,^a^ n (%) (missing = 18)	78 (0.9)	592 (5.6)	**<**.**0001**
ȃImproved drinking water, n (%)	7343 (83.4)	9078 (85.8)	**<**.**0001**
**Clinical variables**			
ȃStunted at enrollment, n (%)	2017 (22.9)	2273 (21.5)	.**018**
ȃDiagnosed pneumonia,^b^ n (%)	120 (1.4)	155 (1.5)	.547
ȃDays to follow-up, mean (SD)	62.34 (9.4)	66.4 (6.9)	**<**.**0001**
ȃDuration of diarrhea,^c^ median (interquartile range)	3.0 (0–7)	2.0 (0–5)	**<**.**0001**

Significant *P* values are shown in bold.

Abbreviation: SD, standard deviation.

Clean fuel includes electric, propane, butane, or natural gas.

Subsequent morbidity information came from a questionnaire completed 2–3 months after enrollment at a home visit.

Tested using the Wilcoxon rank sum test.

#### Analyses

We used data from the GEMS and VIDA matched, case-control studies; however, the outcome of interest presented here was stunting 2–3 months following an episode of MSD. Since the case-control (MSD) status at enrollment is the primary predictor (ie, the exposure variable), we renamed cases as “children with MSD” and controls as “children without MSD.” Demographic (age, sex, and study site), SES, and clinical characteristics (MSD status at enrollment, stunting status at enrollment, intercurrent pneumonia, duration of diarrhea, and days to follow-up) were considered as potential confounders. Four interaction terms were included (MSD status at enrollment × stunting status at enrollment, age group × stunting status at enrollment, study site × stunting status at enrollment, and sex × stunting status at enrollment) due to the heterogeneity of these effects by stunting status at enrollment [11]. Maternal report of facility-diagnosed pneumonia between enrollment and follow-up was included because diarrhea may predispose to pneumonia in undernourished children [12, 13].

Only children with height/length measured at both enrollment and follow-up were included in the analysis. Those with missing values, including those who were lost to follow-up or died, were excluded. No data imputation was performed.

Children's characteristics at enrollment from GEMS were compared with those from VIDA using *χ*^2^ tests for categorical variables and *t* tests for continuous variables. The proportion stunted at enrollment and the proportion stunted at follow-up were calculated by study, MSD status, study site, and age group. Within each study site and age group in the study, a *χ*^2^ test was used to compare the proportion stunted, separately at enrollment and follow-up, between children with MSD and those who were diarrhea-free at enrollment. The McNemar test was used to compare the proportion stunted at enrollment and the proportion stunted at follow-up.

To compare the impact of an episode of MSD on growth between GEMS and VIDA, we used a mixed-effects logistic regression model to estimate the odds of stunting at the follow-up visit while taking into account the correlations among matched cases and controls introduced by the study design. To examine the heterogeneous effects between GEMS and VIDA (ie, before vs after rotavirus vaccine introduction), each predictor listed above including the interaction terms was further interacted with the study indicator (GEMS vs VIDA). In the model, if a higher-order interaction term was included (eg, MSD status at enrollment × stunting status at enrollment × study indicator), all corresponding possible lower-order interactions (eg, MSD status at enrollment × stunting status at enrollment, stunting status at enrollment × study indicator, MSD status at enrollment × study indicator) and the main effects (eg, MSD status at enrollment, stunting status at enrollment, study indicator) were also included (Supplementary Material).

SAS version 9.4 (SAS Institute, Cary, NC) was used for all summary statistics and associated tests, STATA/SE version 17 was used to fit the mixed-effects logistic regression model, and figures were created using R version 3.6.1. Unless otherwise stated, a *P* value < .05 was considered statistically significant.

### Ethics Review

This study was approved by the ethical review committees at the University of Maryland–Baltimore; the Centers for Disease Control and Prevention; The Gambia Government/Medical Research Council/Gambia at the London School of Hygiene & Tropical Medicine; the Comité d'Ethique de la Faculté de Médecine, de Pharmacie, et d'Odonto-Stomatologie, Bamako, Mali; and the Kenya Medical Research Institute Scientific & Ethics Review Unit in Siaya County, Kenya.

## RESULTS

**Figure 1.**
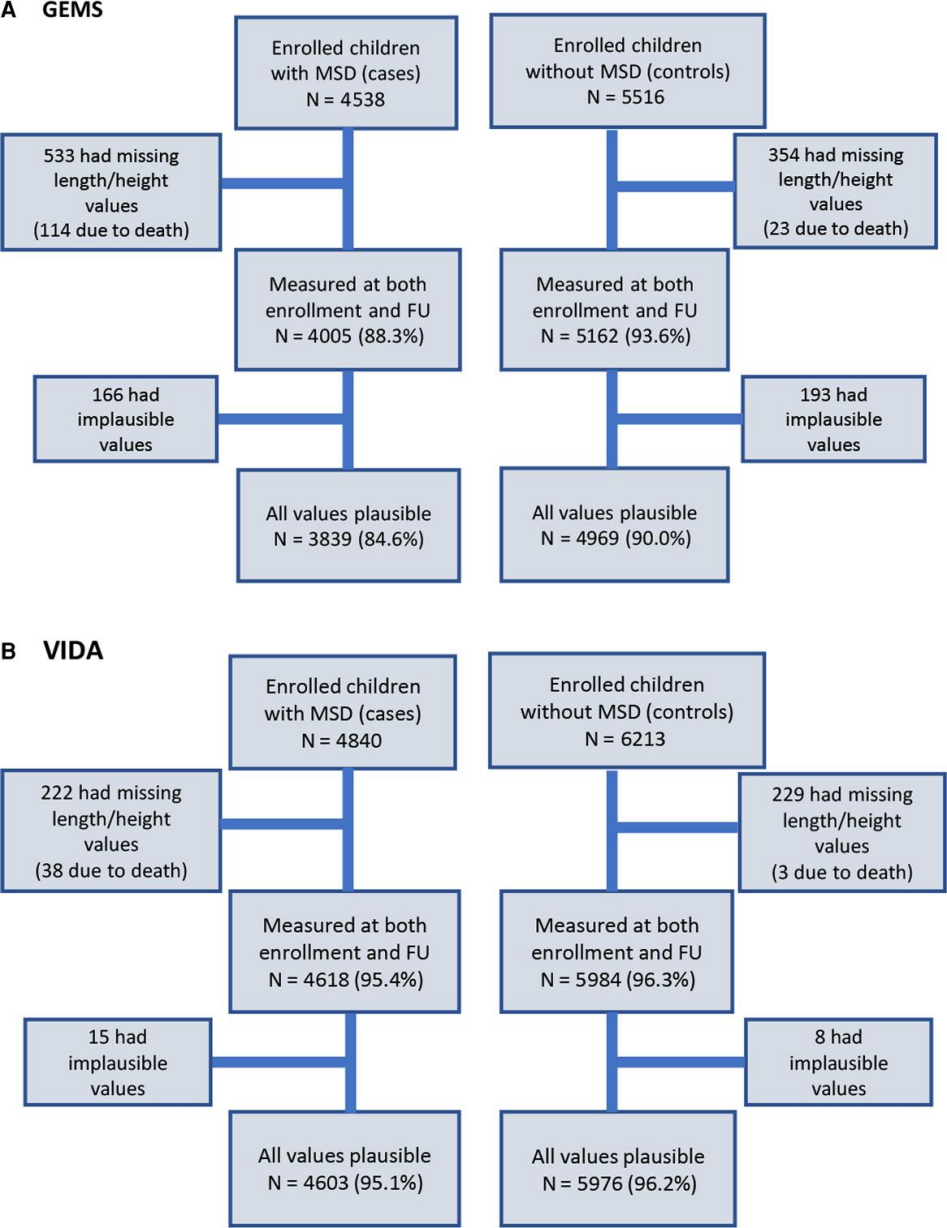
Steps of anthropometric data cleaning to derive analyzable samples in GEMS and VIDA. Abbreviations: FU, follow-up; GEMS, Global Enteric Multicenter Study; VIDA, Vaccine Impact on Diarrhea in Africa study.

A total of 8808 children from GEMS (3839 with MSD and 4969 without MSD) and 10 579 children from VIDA (4603 with MSD and 5976 without MSD) were analyzed after missing height measurements, death, and biologically implausible measurements were removed (Figure 1). No children in GEMS received rotavirus vaccine. In VIDA, among all children who were age-eligible for rotavirus vaccine, 87.5% were fully vaccinated, 8% were partially vaccinated, 1.9% were not vaccinated, and 2.5% had missing vaccination status.

Sociodemographic and clinical differences were observed between GEMS and VIDA as follows (Table 1). VIDA children were more likely than GEMS children to have educated caretakers (34.6% vs 26.1%, *P* < .001) and less likely to have households that were crowded (40.3% vs 42.7%, *P* = .001). VIDA children were more likely to live in a household that had electricity (54.0% vs 44.6%, *P* < .0001), a telephone (96.3% vs 82.4%, *P* < .0001), a television (45.8% vs 43.2%, *P* = .0004), a refrigerator (15.9% vs 13.7%, *P* < .0001), a scooter (50.1% vs 40.9%, *P* < .0001) or a cart (27.7% vs 22.3%, *P* < .0001). Households of VIDA children were more likely to own agricultural land (58.8% vs 51.3%, *P* < .0001), have a finished floor (72.7% vs 67.0, *P* < .0001), use clean cooking fuel (5.6% vs 0.9%, *P* < .0001), and have access to improved drinking water (85.8% vs 83.4%, *P* < .0001). The average duration of an episode of diarrhea was longer for GEMS children compared with VIDA children (median, 3.0; interquartile range [IQR], 0–7 in GEMS vs median, 2.0; IQR, 0–5 in VIDA; *P* < .0001 by Wilcoxon rank sum test).

In both studies, the proportion of children stunted at enrollment increased with age in the 0–11, 12–23, and 24–59 month strata (13.8%, 27.4%, 29.5%, respectively, in GEMS and 14.0%, 25.4%, 25.5% in VIDA, respectively) and at follow-up (19.4%, 30.4%, 28.9% in GEMS and 20.1%, 28.9%, 26.0% in VIDA). In GEMS, among children aged <24 months with MSD and without MSD and across study sites, a significantly higher proportion of children were stunted at follow-up compared with at enrollment with the exception of Malian children aged 12–23 months with MSD who were not more stunted at follow-up compared with at enrollment (Table 2). It is also notable that in the 24–59 month age stratum, stunting was significantly less common at follow-up than at enrollment (both with and without MSD) in Mali, while in Kenya, a significantly higher proportion of children with MSD were stunted at follow-up compared with at enrollment (Table 2). When children with MSD were compared with those without MSD at each site, significant differences were not seen except for children from The Gambia in the 24–59 month age stratum where stunting was more common among those with MSD both at enrollment and at follow-up in GEMS (Table 2). In VIDA, stunting was also more common at follow-up than at enrollment for all children aged <24 months, with the exception of children aged 12–23 month in Mali (Table 3). In comparing children with MSD vs children without MSD in VIDA, significant differences were not seen except for Malian children aged 12–23 months at follow-up, and 24–59 months both at enrollment and follow-up.

**Table 2.** Prevalence of Stunting at Enrollment and Follow-up in Children With and Without Moderate-to-Severe Diarrhea by Site and Age in the Global Enteric Multicenter Study Before Rotavirus Vaccine Introduction

	The Gambia			Mali			Kenya		
	Children With MSD, n (%)	Children Without MSD, n (%)	*P* Value^a^	Children With MSD, n (%)	Children Without MSD, n (%)	*P* Value^a^	Children With MSD, n (%)	Children Without MSD, n (%)	*P* Value^a^
Age, 0–11 m	(n = 324)	(n = 497)		(n = 600)	(n = 636)		(n = 598)	(n = 628)	
ȃStunted at enrollment	47 (14.5)	73 (14.7)	.94	62 (10.3)	54 (8.5)	.27	112 (18.7)	105 (16.7)	.36
ȃStunted at follow-up	67 (20.7)	103 (20.7)	.99	88 (14.7)	71 (11.2)	.07	165 (27.6)	143 (22.8)	.05
ȃ*P* value^b^	**.0009**	**<.0001**		**.0001**	**.0041**		**<.0001**	**<.0001**	
Age, 12–23 m	(n = 354)	(n = 546)		(n = 570)	(n = 631)		(n = 359)	(n = 582)	
ȃStunted at enrollment	92 (26.0)	164 (30.0)	.19	113 (19.8)	127 (20.1)	.90	119 (33.2)	217 (37.3)	.20
ȃStunted at follow-up	126 (35.6)	182 (33.3)	.49	109 (19.1)	129 (20.4)	.57	146 (40.7)	232 (39.9)	.81
ȃ*P* value^b^	**<.0001**	**.0244**		.49	.75		**<.0001**	**.0357**	
Age, 24–59 mo	(n = 133)	(n = 300)		(n = 552)	(n = 578)		(*n* = 349)	(n = 571)	
ȃStunted at enrollment	53 (39.9)	89 (29.7)	.**04**	128 (23.2)	116 (20.1)	.20	127 (36.4)	219 (38.4)	.55
ȃStunted at follow-up	52 (39.1)	83 (27.7)	.**02**	114 (20.7)	99 (17.1)	.13	144 (41.3)	226 (39.6)	.61
ȃ*P* value^b^	.74	.27		**.0060**	**.0004**		**.0016**	.26	

Significant *P* values are shown in bold.

Abbreviation: MSD, moderate-to-severe diarrhea.

*χ* ^2^ test of association between stunting status (yes/no) at enrollment and MSD status (with and without MSD), similarly for stunting status at follow-up.

McNemar test comparing the proportion stunted at enrollment with the proportion stunted at follow-up within each MSD/without MSD group.

**Table 3.** Prevalence of Stunting at Enrollment and Follow-up in Children With and Without Moderate-to-Severe Diarrhea by Site and Age in the Vaccine Impact on Diarrhea in Africa Study After Rotavirus Vaccine Introduction

	The Gambia			Mali			Kenya		
	Children With MSD, n (%)	Children Without MSD, n (%)	*P* Value^a^	Children With MSD, n (%)	Children Without MSD, n (%)	*P* Value^a^	Children With MSD, n (%)	Children Without MSD, n (%)	*P* Value^a^
Age, 0–11 m	(n = 512)	(n = 670)		(n = 562)	(n = 665)		(n = 555)	(n = 700)	
ȃStunted at enrollment	82 (16.0)	100 (14.9)	.66	52 (9.3)	59 (8.9)	.90	102 (18.4)	119 (17.0)	.57
ȃStunted at follow-up	128 (25.0)	150 (22.4)	.33	84 (14.9)	84 (12.6)	.27	140 (25.2)	152 (21.7)	.16
*ȃP* value^b^	**<.0001**	**<.0001**		**<.0001**	**.0006**		**<.0001**	**<.0001**	
Age, 12–23 m	(n = 581)	(n = 715)		(n = 532)	(n = 620)		(n = 505)	(n = 703)	
ȃStunted at enrollment	161 (27.7)	215 (30.1)	.38	104 (19.5)	99 (16.0)	.13	141 (27.9)	208 (29.6)	.57
ȃStunted at follow-up	194 (33.4)	238 (33.3)	1.00	124 (23.3)	102 (16.5)	.**004**	171 (33.9)	229 (32.6)	.68
*ȃP* value^b^	**<.0001**	**.002**		**.002**	**.75**		**<.0001**	**.004**	
Age, 24–59 m	(n = 486)	(n = 661)		(n = 448)	(n = 611)		(n = 422)	(n = 631)	
ȃStunted at enrollment	169 (34.8)	207 (31.3)	.24	80 (17.9)	78 (12.8)	.**03**	112 (26.5)	185 (29.3)	.36
ȃStunted at follow-up	178 (36.6)	213 (32.2)	.14	78 (17.4)	77 (12.6)	.**04**	121 (28.7)	181 (28.7)	1.00
*ȃP* value^b^	.16	.36		.83	1.00		.066	.48	

Significant *P* values are shown in bold.

Abbreviation: MSD, moderate-to-severe diarrhea.

*χ* ^2^ test of association between stunting status (yes/no) at enrollment and MSD status (with and without MSD), similarly for stunting status at follow-up.

McNemar test comparing the proportion stunted at enrollment with the proportion stunted at follow-up within each MSD/without MSD group.

The summary of the mixed-effects logistic regression model is included in the Supplementary Material, and the estimated effects of interest are reported in Table 4. Among children who were not stunted at enrollment, an episode of MSD significantly increased the odds of being stunted at follow-up by 31% in GEMS (adjusted odds ratio [aOR], 1.31; 95% confidence interval [CI]: 1.04–1.64; *P* = .023) and by 30% in VIDA (aOR, 1.30; 95% CI: 1.04–1.61; *P* = .019), after controlling for other factors. There was no difference in the magnitude of this association between the 2 studies (*P* = .965). In contrast, among those already stunted at enrollment, an episode of MSD did not significantly affect the odds of being stunted at follow-up in either GEMS or VIDA, and there was no difference in the effect between the 2 studies (*P* = .948).

**Table 4.** Comparison of the Effect of Each Predictor on the Odds of Stunting (HAZ <−2) at Follow-up Between the Global Enteric Multicenter Study and the Vaccine Impact on Diarrhea in Africa Study (Before and After Rotavirus Vaccine Introduction) Using the Mixed Effects Logistic Regression Model (see Supplementary Material)

Variables	GEMS		VIDA		GEMS vs VIDA *P* Value
	aOR^a^ (95% CI)	*P* Value	aOR^a^ (95% CI)	*P* Value	
**Children not stunted at enrollment**					
ȃChildren without MSD	1		1		
ȃChildren with MSD	1.31 (1.04–1.64)	**.023**	1.30 (1.04–1.61)	.**019**	.965
ȃȃAge stratum, m					
ȃȃȃ24–59	1		1		
ȃȃȃ0–11	1.89 (1.42–2.50)	**<.001**	2.64 (2.03–3.44)	**<**.**001**	.085
ȃȃȃ12–23	2.08 (1.56–2.79)	**<.001**	2.25 (1.72–2.95)	**<**.**001**	.704
ȃȃSite					
ȃȃȃMali	1		1		
ȃȃȃThe Gambia	2.30 (1.57–3.36)	**<.001**	1.17 (0.81–1.70)	.40	.**014**
ȃȃȃKenya	2.54 (1.63–3.94)	**<.001**	1.10 (0.72–1.67)	.66	.**007**
ȃȃMale vs female	1.22 (1.00–1.47)	**.045**	1.38 (1.15–1.66)	**<**.**001**	.333
**Children stunted at enrollment**					
ȃChildren without MSD	1		1		
ȃChildren with MSD	1.03 (0.76–1.41)	0.85	1.02 (0.72–1.43)	.93	.95
ȃȃAge stratum, m					
ȃȃȃ24–59	1		1		
ȃȃȃ0–11	0.84 (0.58–1.22)	.37	0.86 (0.56–1.31)	.48	.95
ȃȃȃ12–23	1.01 (0.74–1.40)	.93	1.00 (0.69–1.44)	1.00	.96
ȃȃSite					
ȃȃȃMali	1		1		
ȃȃȃThe Gambia	0.95 (0.62–1.47)	.82	1.40 (0.87–2.25)	.16	.24
ȃȃȃKenya	1.74 (1.07–2.85)	**.026**	1.93 (1.14–3.26)	.**014**	.78
ȃȃMale vs female	1.10 (0.83–1.47)	.51	0.96 (0.69–1.33)	.79	.53
>2 vs ≤2 children aged <5 y per caretaker	1.08 (0.88–1.33)	.47	1.32 (1.08–1.61)	**.008**	.18
Crowding (>3 vs ≤3 people sleeping per room)	1.03 (0.88–1.21)	.69	1.05 (0.90–1.23)	.54	.88
Caretaker with ≥ vs < primary school education	0.83 (0.68–1.02)	.078	0.81 (0.67–0.99)	**.035**	.87
Household possessions					
ȃCar (vs no car)	1.06 (0.8–1.4)	.67	0.89 (0.68–1.18)	.43	.39
ȃScooter (vs no scooter)	0.98 (0.78–1.24)	.87	1.08 (0.88–1.32)	.46	.54
ȃBike (vs no bike)	1 (0.83–1.2)	.98	0.95 (0.79–1.14)	.58	.71
ȃCart (vs no cart)	0.92 (0.69–1.21)	.55	0.99 (0.76–1.28)	.93	.70
ȃPhone (vs no phone)	1 (0.8–1.25)	.98	1.09 (0.71–1.67)	.71	.73
ȃRadio (vs no radio)	0.82 (0.65–1.04)	.10	0.92 (0.76–1.12)	.39	.49
ȃAgricultural land (vs no land)	1.42 (1.08–1.86)	**.011**	1.21 (0.9–1.62)	.22	.42
Principal component electricity, television, refrigerator	1.01 (0.93–1.1)	.81	0.91 (0.85–0.99)	**.024**	.088
Household with finished vs unfinished floor	0.98 (0.76–1.26)	.85	0.92 (0.73–1.15)	.46	.72
Household uses clean vs unclean cooking fuel	0.72 (0.27–1.91)	.51	0.61 (0.4–0.95)	**.030**	.77
Improved vs unimproved drinking water source	0.84 (0.68–1.04)	.11	0.82 (0.65–1.03)	.088	.90
History of pneumonia before follow-up visit^b^	2.31 (1.31–4.06)	**.004**	1.12 (0.62–2.01)	.71	.081
Days to follow-up	1.01 (1–1.02)	.078	1.03 (1.02–1.04)	**<.001**	.**002**
Duration of diarrhea, d	1.01 (0.99–1.03)	.45	1.02 (0.99–1.04)	.16	.61

Significant *P* values are shown in bold.

Abbreviations: aOR, adjusted odds ratio; CI, confidence interval; GEMS, Global Enteric Multicenter Study; MSD, moderate-to-severe diarrhea; VIDA, Vaccine Impact on Diarrhea in Africa study.

aOR based on the mixed-effects logistic regression model.

Follow-up visit was performed at home 2–3 months after enrollment.

Among children not stunted at enrollment, those belonging to the 0–11 and 12–23 month age strata were more likely to be stunted at follow-up compared with children aged 24–59 months in both studies (Table 4). Infants aged 0–11 months in VIDA had a higher odds of subsequent stunting compared with infants in GEMS, although the difference was not statistically significant (aOR, 2.64; 95% CI: 2.03–3.44 in VIDA vs aOR, 1.89; 95% CI: 1.42–2.50 in GEMS; *P* = .085). Compared with Malian children, children from The Gambia and Kenya had higher odds of stunting at follow-up in both studies, but the site effect was significant in GEMS only, and the strength of association was significantly higher in GEMS compared with VIDA. Males had a significantly higher odds of being stunted at follow-up compared with females in both studies (aOR, 1.22; 95% CI: 1.00–1.47 in GEMS and aOR, 1.38; 95% CI: 1.15–1.66 in VIDA), which was not significantly different between studies (*P* = .33).

Among children who were stunted at enrollment, children from Kenya had a significantly higher odds of stunting at follow-up compared with children from Mali in both studies (aOR, 1.93; 95% CI: 1.14–3.26; *P* = .014 in VIDA vs aOR, 1.74; 95% CI: 1.07–2.85; *P* = .026 in GEMS; Table 4), and the strength of association was not significantly different between the studies (*P* = .78).

Other variables that significantly increased the odds of stunting at follow-up included a longer duration of follow-up, caretakers with >2 children aged <5 years under their supervision in VIDA, a history of pneumonia in GEMS, and having agricultural land in GEMS. Caretakers with primary education or higher and the use of clean cooking fuel in VIDA significantly decreased the odds of stunting at follow-up (Table 4).

## DISCUSSION

The findings presented here from 3 sites in sub-Saharan Africa support the hypothesis that rotavirus vaccine introduction did not significantly alter the impact of MSD on stunting. The corollary is that enteric pathogens other than rotavirus had more impact on stunting, in particular, *Cryptosporidium*, *Shigella*, and typical EPEC and ETEC [3, 14–16], for which vaccine introduction had no measurable impact [4].

Examining the risk of stunting following an episode of MSD after rotavirus vaccine introduction using data from GEMS (before vaccine introduction) and VIDA (after vaccine introduction), we found that, among children who were not stunted at baseline, an episode of MSD increased the odds of stunting during the 2–3 months after the episode in both studies (31% in GEMS and 30% in VIDA). The magnitude of the association did not differ between the 2 studies. These findings emphasize the significant effect of an episode of MSD on the short-term occurrence of stunting and highlight the persistence of this threat despite the rollout of vaccines against what had been the leading diarrheal pathogen among children aged <5 years. The proportion of MSD attributable to rotavirus in The Gambia, Mali, and Kenya was reduced by 25.1%, 35%, and 36.9% among infants aged 0–11 months and by 10.6%, 0%, and 16.2% among toddlers aged 12–23 months, respectively, after the introduction of rotavirus vaccine [4], yet the strong association between MSD and subsequent development of stunting remains unchanged.

Among children who were stunted at enrollment, those from Kenya had a significantly higher odds of stunting at follow-up compared with those from Mali in both studies. Kenya did not have a higher AF of pathogens associated with stunting or more sociodemographic risk factors [4]. One possible explanation is the burden of human immunodeficiency virus (HIV) in Kenya, which may have augmented baseline stunting and created a subset of children who are more vulnerable to stunting following enteric infection. Of Kenya's 47 counties, Siaya, the location of GEMS and VIDA, had the highest HIV prevalence (21% in those aged >15 years) during the years that VIDA was conducted [17], while Mali and The Gambia have maintained low HIV prevalence rates [18].

To date, evidence has been lacking on the effect of rotavirus vaccine introduction on diarrhea-mediated growth faltering. Two studies examined the impact of vaccine on linear growth. A positive impact of vaccine was suggested in a cross-sectional study that used data from a national survey in Peru conducted when rotavirus vaccine coverage was approximately 75%. Children aged 6–60 months who received rotavirus vaccine had a mean height for age that was 0.06 standard deviations higher than for unvaccinated children [19]. On the other hand, a post hoc analysis of Bangladeshi children who participated in a phase 3 trial of pentavalent rotavirus vaccine found no difference in HAZ between vaccine and placebo recipients at an anthropometry follow-up 1 year after the trial [20].

An important finding in our study was that higher levels of maternal education and higher SES were protective against stunting, which is consistent with previous studies [21]. These findings emphasize the importance of addressing growth-faltering prevention in a wholistic fashion that includes the sociodemographic vulnerabilities in the child's environment as well as pathogen exposures.

Several limitations of our study are noteworthy. Data collection was limited to 3 sites in sub-Saharan Africa, so the broader generalizability cannot be determined. Although we were able to assess a broad spectrum of confounders, there may be others that we could not assess, such as feeding practices, dietary diversity, maternal health and nutrition, impact of HIV, and nondiarrheal infections, that can increase risk of stunting. Each enrolled child underwent a single follow-up visit 2–3 months after enrollment. This permitted a precise assessment of vital status and interim growth but did not enable prospective assessment of interim infections or other morbid events. The strengths of the study include the large sample size and the ability to compare prospectively collected data at the same sites using the same methodology over a 10-year period among children living in sub-Saharan Africa where diarrheal morbidity and mortality are high.

## CONCLUSIONS

Rotavirus vaccines substantially reduce diarrheal disease and mortality and are cost-effective, including in low- and middle-income countries [22]. Our findings indicate that the remaining burden of medically attended diarrhea is strongly driven by subsequent stunting among children aged <5 years in sub-Saharan Africa and is likely to be related to enteric pathogens other than rotavirus that have more impact on stunting. These findings identify a compelling need for a wholistic approach that is focused on strategies to prevent and manage specific pathogens that cause childhood stunting.

## Supplementary Data

Supplementary materials are available at *Clinical Infectious Diseases* online. Consisting of data provided by the authors to benefit the reader, the posted materials are not copyedited and are the sole responsibility of the authors, so questions or comments should be addressed to the corresponding author.

## Supplementary Material

ciac948_Supplementary_Data

## Notes

***Acknowledgments.*** We express our deep gratitude to the families who participated in both the Global Enteric Multicenter Study (GEMS) and the Vaccine Impact on Diarrhea in Africa study (VIDA), the clinical and field staff for their exceptional hard work and dedication, and the physicians and administration at each site who generously provided facilities for the conduct of the study. We are indebted to all the investigators involved in GEMS. We thank Irwin Shorr for providing training in anthropometry in GEMS and to the data management team involved in GEMS (Perry Point VA Medical Center) and VIDA (the Emmes Company, LLC) for expert data management and reporting. Special thanks go to Myron M. Levine for oversight of GEMS; Carl Kirkwood, Duncan Steele, and Anita Zaidi at the Bill & Melinda Gates Foundation for helpful oversight; Kathy Neuzil for thoughtful suggestions; and the following members of our International Scientific Advisory Committee for providing insightful comments and guidance: Janet Wittes (chair), George Armah, John Clemens, Christopher Duggan, Stephane Helleringer, Ali Mokdad, James Nataro, and Halvor Sommerfelt.

***Supplement sponsorship.*** This supplement was sponsored by the Bill & Melinda Gates Foundation. This study is based on research funded in part by grants from the Bill & Melinda Gates Foundation (OPP1111236/OPP1116751).

***Financial support.*** Both the GEMS and VIDA studies were funded by the Bill & Melinda Gates Foundation (GEMS grant 38874 and VIDA grant OPP1111236 and grant OPP1116751).
