# Supplementary material for: Stunting Following Moderate-to-Severe Diarrhea Among Children Aged <5 Years in Africa Before and After Rotavirus Vaccine Introduction: A Comparison of the Global Enteric Multicenter Study and the Vaccine Impact on Diarrhea in Africa (VIDA) Study
Source: Clin Infect Dis. 2023 Apr 19;76(Suppl 1):S49–57. doi: 10.1093/cid/ciac948 (PMC10116564; doi:10.1093/cid/ciac948)
Supplement: ciac948_Supplementary_Data [file ciac948_supplementary_data.docx]

**APPENDIX**

**Stunting following moderate-to-severe diarrhea among children younger 5 years in Africa before and after rotavirus vaccine introduction: A comparison of GEMS and VIDA**

Dilruba Nasrin, Yuanyuan Liang, Jennifer R. Verani, Helen Powell, Samba Sow, Richard Omore, M. Jahangir Hossain, Sanogo Doh, Syed M.A. Zaman, Joquina Chiquita M. Jones, Alex Awuor**,** Irene N. Kasumb, Ben Ochieng^7^, Sharon M Tennant, Usha Ramakrishnan, Karen L. Kotloff

**Appendix: Summary of the mixed-effects logistic regression model with all predictor variables and interaction terms**

| **END_STUNT_BIN** | **Odds ratio** | **[95% confidence interval]** | | **P>z** |
| --- | --- | --- | --- | --- |
|  |  |  |  |  |
| stunted at enrollment (Yes vs. No) | 325.69 | 190.6866 | 556.2735 | 0 |
| MSD status at enrollment (MSD vs. without MSD) | 1.31 | 1.038338 | 1.644115 | 0.023 |
|  |  |  |  |  |
| Agegroup (reference=3) |  |  |  |  |
| 1 | 1.89 | 1.421916 | 2.501493 | <0.001 |
| 2 | 2.08 | 1.558125 | 2.787269 | <0.001 |
|  |  |  |  |  |
| Site (reference=2) |  |  |  |  |
| 1 | 2.30 | 1.56795 | 3.36221 | <0.001 |
| 3 | 2.54 | 1.634136 | 3.942895 | <0.001 |
|  |  |  |  |  |
| Gender (Male vs. female) | 1.22 | 1.004436 | 1.470605 | 0.045 |
|  |  |  |  |  |
| Stunted at enrollment × MSD status |  |  |  |  |
| 1 1 | 0.79 | 0.5615214 | 1.109334 | 0.173 |
|  |  |  |  |  |
| Stunted at enrollment × Agegroup |  |  |  |  |
| 1 1 | 0.45 | 0.2814388 | 0.7120621 | 0.001 |
| 1 2 | 0.49 | 0.3159348 | 0.7497048 | 0.001 |
|  |  |  |  |  |
| Stunted at enrollment × site |  |  |  |  |
| 1 1 | 0.41 | 0.269292 | 0.637382 | <0.001 |
| 1 3 | 0.69 | 0.4549774 | 1.038441 | 0.075 |
|  |  |  |  |  |
| Stunted at enrollment × gender |  |  |  |  |
| 1 1 | 0.91 | 0.64238 | 1.277941 | 0.574 |
|  |  |  |  |  |
| 2.study | 0.22 | 0.0643332 | 0.7335774 | 0.014 |
|  |  |  |  |  |
| Stunted at enrollment × study |  |  |  |  |
| 1 2 | 1.33 | 0.651573 | 2.708642 | 0.435 |
|  |  |  |  |  |
| MSD status × study |  |  |  |  |
| 1 2 | 0.99 | 0.7232569 | 1.363081 | 0.965 |
|  |  |  |  |  |
| agegroup × study |  |  |  |  |
| 1 2 | 1.40 | 0.9540464 | 2.05943 | 0.085 |
| 2 2 | 1.08 | 0.7259657 | 1.606971 | 0.704 |
|  |  |  |  |  |
| site × study |  |  |  |  |
| 1 2 | 0.51 | 0.2998409 | 0.8713149 | 0.014 |
| 3 2 | 0.43 | 0.2360587 | 0.7937081 | 0.007 |
|  |  |  |  |  |
| gender × study |  |  |  |  |
| 1 2 | 1.139 | 0.8752086 | 1.481925 | 0.333 |
|  |  |  |  |  |
| Stunted at enrollment × MSD status × study |  |  |  |  |
| 1 1 2 | 0.99 | 0.6011272 | 1.636653 | 0.975 |
|  |  |  |  |  |
| Stunted at enrollment × agegroup × study |  |  |  |  |
| 1 1 2 | 0.73 | 0.3685477 | 1.427542 | 0.353 |
| 1 2 2 | 0.91 | 0.4876269 | 1.708854 | 0.776 |
|  |  |  |  |  |
| Stunted at enrollment × site × study |  |  |  |  |
| 1 1 2 | 2.88 | 1.555064 | 5.327094 | 0.001 |
| 1 3 2 | 2.56 | 1.375939 | 4.755887 | 0.003 |
|  |  |  |  |  |
| Stunted at enrollment × gender × study |  |  |  |  |
| 1 1 2 | 0.76 | 0.4590947 | 1.268986 | 0.298 |
|  |  |  |  |  |
| Caretaker with at least primary school education (vs. less than primary education) | 0.83 | 0.6755713 | 1.020787 | 0.078 |
| FUEL_CLEAN | 0.72 | 0.2713611 | 1.914987 | 0.511 |
| IMPROV_WATER_BIN | 0.84 | 0.6760337 | 1.038256 | 0.106 |
| FINISH_FL | 0.98 | 0.7552155 | 1.259109 | 0.847 |
| crowded | 1.03 | 0.8807158 | 1.210696 | 0.693 |
| CHILD_CARE | 1.08 | 0.878451 | 1.32549 | 0.468 |
| HOUSE_BIKE | 0.99 | 0.8272753 | 1.202215 | 0.977 |
| HOUSE_PHONE | 0.99 | 0.7958205 | 1.249513 | 0.98 |
| HOUSE_CAR | 1.06 | 0.8032996 | 1.40403 | 0.673 |
| HOUSE_CART | 0.92 | 0.6935511 | 1.214958 | 0.549 |
| HOUSE_SCOOT | 0.98 | 0.7788844 | 1.235404 | 0.87 |
| HOUSE_RADIO | 0.82 | 0.6541369 | 1.040136 | 0.104 |
| HOUSE_AGLAND | 1.42 | 1.084971 | 1.856818 | 0.011 |
| pc1_ETF | 1.01 | 0.9292267 | 1.09839 | 0.811 |
| Pneumonia | 2.31 | 1.314245 | 4.055009 | 0.004 |
| Durdays | 1.01 | 0.999046 | 1.018338 | 0.078 |
| DUR_DRH | 1.01 | 0.9868718 | 1.030036 | 0.453 |
|  |  |  |  |  |
| Caretaker with at least primary school education (vs. less than primary education) × study |  |  |  |  |
| 1 2 | 0.98 | 0.7352064 | 1.296569 | 0.869 |
|  |  |  |  |  |
| FUEL_CLEAN × study |  |  |  |  |
| 1 2 | 0.85 | 0.2918108 | 2.488572 | 0.77 |
|  |  |  |  |  |
| IMPROV_WATER_BIN × study |  |  |  |  |
| 1 2 | 0.98 | 0.7169065 | 1.338815 | 0.898 |
|  |  |  |  |  |
| FINISH_FL × study |  |  |  |  |
| 1 2 | 0.94 | 0.6651036 | 1.325827 | 0.721 |
|  |  |  |  |  |
| Crowded × study |  |  |  |  |
| 1 2 | 1.02 | 0.812413 | 1.274536 | 0.879 |
|  |  |  |  |  |
| CHILD_CARE × study |  |  |  |  |
| 1 2 | 1.22 | 0.9142236 | 1.63003 | 0.176 |
|  |  |  |  |  |
| HOUSE_BIKE × study |  |  |  |  |
| 1 2 | 0.95 | 0.7308082 | 1.239632 | 0.714 |
|  |  |  |  |  |
| HOUSE_PHONE × study |  |  |  |  |
| 1 2 | 1.09 | 0.6711366 | 1.766807 | 0.73 |
|  |  |  |  |  |
| HOUSE_CAR × study |  |  |  |  |
| 1 2 | 0.84 | 0.5664521 | 1.248855 | 0.391 |
|  |  |  |  |  |
| HOUSE_CART × study |  |  |  |  |
| 1 2 | 1.08 | 0.7357959 | 1.575742 | 0.703 |
|  |  |  |  |  |
| HOUSE_SCOOT × study |  |  |  |  |
| 1 2 | 1.10 | 0.8103221 | 1.495424 | 0.539 |
|  |  |  |  |  |
| HOUSE_RADIO × study |  |  |  |  |
| 1 2 | 1.11 | 0.8224686 | 1.507463 | 0.487 |
|  |  |  |  |  |
| HOUSE_AGLAND × study |  |  |  |  |
| 1 2 | 0.85 | 0.5698217 | 1.265405 | 0.422 |
|  |  |  |  |  |
| Study × pc1_ETF |  |  |  |  |
| 2 | 0.91 | 0.8081265 | 1.014847 | 0.088 |
|  |  |  |  |  |
| Pneumonia × study |  |  |  |  |
| 1 2 | 0.48 | 0.2139977 | 1.092713 | 0.081 |
|  |  |  |  |  |
| Study × durdays |  |  |  |  |
| 2 | 1.02 | 1.008334 | 1.037814 | 0.002 |
|  |  |  |  |  |
| Study × DUR_DRH |  |  |  |  |
| 2 | 1.01 | 0.9773366 | 1.040001 | 0.607 |

Study: 1=GEMS, 2=VIDA

MSD status: 0=without MSD, 1=with MSD

agegroup: 1=0-11, 2=12-23, 3=24-59 months

site: 1=Gambia, 2=Mali, 3=Kenya

gender: 1=Male, 2=Female

FUEL_CLEAN: Household uses clean vs unclean cooking fuel

IMPROV_WATER_BIN: Improved vs unimproved drinking water source

FINISH_FL: Household with finished vs unfinished floor

crowded: Crowding (>3 vs <=3 people sleeping per room)

CHILD_CARE: >2 vs <=2 children under 5 years per caretaker

HOUSE_BIKE: Bike (vs no bike)

HOUSE_PHONE: Phone (vs no phone)

HOUSE_CAR: Car (vs no car)

HOUSE_CART: Cart (vs no cart)

HOUSE_SCOOT: Scooter (vs. no scooter)

HOUSE_RADIO: Radio (vs no radio)

HOUSE_AGLAND: Agricultural land (vs. no land)

pc1_ETF: Principal component ETR (electricity, television, refrigerator)

Pneumonia: History of pneumonia before ~60 days follow-up

Durdays: Days to follow-up, day

DUR_DRH: Duration of diarrhea, day
